# Health-related consequences of the type and utilization rates of electronic devices by college students

**DOI:** 10.1186/s12889-021-11975-3

**Published:** 2021-11-01

**Authors:** Mark Benden, Ranjana Mehta, Adam Pickens, Brett Harp, Matthew Lee Smith, Samuel D. Towne, S. Camille Peres

**Affiliations:** 1 ErgoCenter, Department of Environmental and Occupational Health, School of Public Health, Texas A&M University, College Station, TX USA; 2 Wm Michael Barnes ‘64 Department of Industrial and Systems Engineering, Texas A&M University, College Station, TX USA; 3 Department of Environmental and Occupational Health, School of Public Health, Texas A&M University, College Station, TX 77843 USA; 4 Center for Population Health and Aging, Texas A&M University, College Station, TX USA; 5 School of Global Health Management and Informatics, University of Central Florida, 4364 Scorpious Street, HPA II, Orlando, FL 32816-2205 USA; 6 Disability, Aging, and Technology Cluster, University of Central Florida, Orlando, Florida 32816-2205 USA; 7 Southwest Rural Health Research Center, Texas A&M University, College Station, TX 77843 USA

**Keywords:** Student, Ergonomics, Posture, Comfort, Sedentary, Smartphone, Tablet

## Abstract

**Background:**

College students are leading an evolution of device use both in the type of device and the frequency of use. They have transitioned from desktop stations to laptops, tablets, and especially smartphones and use them throughout the day and into the night.

**Methods:**

Using a 35-min online survey, we sought to understand how technology daily usage patterns, device types, and postures affect pain and discomfort to understand how knowledge of that pain might help students avoid it. Data were analyzed from 515 students (69.5% male) who completed an internet-delivered survey (81.3% response rate).

**Results:**

Participants ranked smartphones as their most frequently used technology (64.0%), followed by laptops and tablets (both 53.2%), and desktop computers (46.4%). Time spent using smartphones averaged over 4.4 h per day. When using their devices, students were more likely to adopt non-traditional workplace postures as they used these devices primarily on the couch or at a chair with no desk.

**Conclusion:**

Recent trends in wireless academic access points along with the portability of small handheld devices, have made smartphones the most common link to educational materials despite having the least favorable control and display scenario from an ergonomic perspective. Further, the potential impact of transitions in work environments due to COVID-19 may further exacerbate ergonomic issues among millions highlighting the need for such work to be carried out.

## Introduction

College students, many of whom are future office workers, have higher levels of screen time and utilize multiple devices at higher rates compared to previous generations [1]. These have resulted in the increases in computer-related musculoskeletal disorders in this specific age group [2]. Studies have shown laptops to be ubiquitous with the college experience [3] and multiple studies have found elevated musculoskeletal discomfort associated with awkward postures for laptop use among laptop-using college students [4]. In some studies—where the prevalence of poor postures was measured across different devices such as tablets, cell phones—laptops and tablets were found to be the worst in terms posture and comfort [5]. Several studies have also explored the use of laptops and tablets as it pertains to discomfort. In those studies, discomfort from laptop and tablet use were clearly connected to poor posture and musculoskeletal symptoms [6, 7]. Studies have reported adoption of poor or awkward postures in conjunction with electronic devices, such as a preference to sit without support when using mobile devices or bending head/neck to view small-screen devices, have led to increased reports of musculoskeletal pain [8]. However, there are currently some important gaps in this literature particularly given the trends showing movement in the direction of increased smartphone use [9]. Further, smartphone ownership among Americans reached 85% versus 77% for devices such as laptops or desktops and 53% for tablet computers according to a recent Pew Research Report [10]. Further, it is necessary to understand those trends from an ergonomic risk perspective. Specifically it is important to understand for college student device use: a) any connection of the rising use of smartphones with respect to posture and self-reported musculoskeletal pain; and b) contextualize smartphone posture and pain relative to laptops, tablets, and desktops.

### Broad implications for public health

While identifying all relevant public health implications associated with poor ergonomic behaviors is outside the scope of this study, given wide variation across settings, populations, and possible health outcomes, we provide some brief examples here. For example, one study investigating musculoskeletal-related problems among students in medical science found high rates of lab-related musculoskeletal issues with the lower back reported as the most common site of issues in the past year [11]. While much broader in terms of work settings and geography, one example investigating lower back pain found the estimated global burden associated with lower back pain (occupationally related) was 21.7 million Disability-Adjusted Life Years (DALYs) [12] in 2010. While this is provided as an example of the potential global burden associated with certain ergonomic-related risk factors, these risk factors and the associated burden can vary as do potential solutions.

### Some potential solutions

In terms of potential solutions to certain ergonomic-related risks, one study investigating ways to reduce the musculoskeletal burden of workers found that ergonomic intervention implementation was associated with reductions in both severe and frequent pain [13]. Further, one somewhat recent review found limited evidence of effective ergonomic interventions from 15 randomized controlled trials, though their inclusion criteria may not represent all settings, interventions, target populations, or location of musculoskeletal disorders (e.g., focused on upper limb and neck) [14]. The authors of this review indicated further need of high-quality research into potential interventions in office settings, among other findings. Furthermore, more general considerations, though not necessarily exhaustive, are worth mentioning.

In general terms, utilization of 1) an optimized control device for data entry, 2) a visual display of that data, and 3) having the display located at an optimal viewing distance, angle, height, and luminance has become the ergonomic standard for most professional work. Translated to what is commercially available today we would describe that optimum control/display product as an ergonomic keyboard and separate mouse along with a well-positioned and high-quality monitor. Monitor size and multiple monitors has begun to erode that theoretical optimum but generally, these combinations are considered more beneficial to user health and safety than the next major step down in ergonomics, a laptop with integrated mouse. Since in this device the displays and controls are connected, one must compromise position of one over the other. Additionally, size of both and design limitations make these combinations more of an occasional use and or portable/travel use device. Finally, handheld tablets and then smartphones are considered the lowest level of ergonomics since once again, the control and display are integrated but, in this case, they are on the same plane and in the truest sense, the display is the control. For each part of portability and weight positives users gain through this progression, they give up on ergonomics, comfort, etc. In the final level of smartphones, they even provide the physical support for the device during use. In classic human factors or industrial engineering terms, this wasteful use of muscle energy is unproductive work that leads to fatigue and then altered and later poor postures with increased use. To further complicate the challenge of providing ideal ergonomic environments is the disruption linked to the dramatic shift of both university education and previously office-based work environments of major industries transitioning, in some cases for many months, to remote working conditions. This may force individuals to rely on their homes to serve as office-like environments, which may not be properly resourced.

In addition to traditional ergonomic risk factors, such as awkward posture and repetitive behavior, non-physical factors, such as cognitive overload or stress, have shown to impact biomechanical and behavioral outcomes, across a variety of activities, ranging from typing [15] to walking [16]. For example, the Ecological Model of MSD [17] proposes that combined physical and psychological strain may affect the detection, labeling, and attribution of sensations arising as the result of biomechanical strain as well as the behavioral response to symptoms. Additional mental demand and/or mental stress have been shown to adversely influence upper extremity muscle activity [18–20], which over a prolonged period of time, can lead to the development of MSDs [21]. It is thus likely that the risk of prolonged usage of electronic devices, which imposes significant muscle activity due to sustained and awkward postures, may be compounded under stress originating from a variety of sources.

### Aims

This study seeks to build on knowledge of college student device use and to further connect it to the rising use of smartphones with respect to posture and musculoskeletal self-reports of pain and to put those smartphone sources in context relative to laptops, tablets, and desktops. Our intention with this study was to first understand this backdrop of ergonomics as it relates to devices and then to quantify how much time college students with all three types of devices spend on each user scenario and how they felt it impacted their body.

## Methods

### Participants

Participants were recruited from a large southwestern tier-1 land-grant university’s geosciences department during the Fall semester as part of a baseline assessment before an ergonomics training program. Upon receipt of administrative information for the semester from the department, all participants were requested to complete a survey online (created in Qualtrics®). Of the 631 students in the program (495 undergraduate and 136 graduate), 515 fully completed the survey (81.3% response rate). The study was approved by the University Institutional Review Board and all methods were performed in accordance with the relevant guidelines and regulations.

### Procedures

The survey distributed to participants was developed by the investigators and contained questions regarding several topic areas on ergonomics knowledge, technology use, and health. This paper, and the items covered herein, will focus on the: a) technology participants used regularly and how much they used that technology; b) postures participants adopted with that technology; and c) amount of pain or discomfort participants were currently experiencing. As mentioned previously, the survey was distributed to participants during the beginning of the Fall 2014 semester before students had participated in ergonomic training programs. The survey was conducted online and took approximately 35 min to complete. No incentives were provided to complete the survey.

### Measures

#### Device usage

Participants responded to several sets of questions created for this study to determine the types of interactive technology (including a control and a display) they used on a regular basis and the level to which they used this technology. The question lines were as follows: “What types of interactive technology devices do you use on a regular basis (e.g., most days of the week?) Check all that apply. (*Tech use*)”; “What types of interactive technology devices do you use most often? Click & Drag to arrange in order. (*Tech most*)”*;* “What % of the time are you interacting with your device: (Note: 0-100%. Please ensure this total to 100% combined device time). (*Tech for*)”*;* “On average, on any given day, how many minutes do you spend interacting with the following device(s) continuously without a break? *(Tech no break*)”; “On average, how many hours do you spend interacting with the following device(s) each day? (There were clarifying instructions regarding the term “interacting”.) (*Hours tech*)”. It should be noted that for those who indicated they were gamers, they were asked to indicate how many hours they spent per day gaming as well. Students self-identified their “gamer” status as this term was identified by student researchers as commonplace among college students. For the study’s purposes, the term generally refereed to those spending multiple hours per week in competitive computer games. Each of the questions required the participants to respond based on five different categories: Tablet, Desktop, Laptop, Smartphone, and Other (participants could indicate what the Other category represented). Finally, participants were asked to indicate the percent of time they used any technology (*Tech for)* for the following activity: School, Work, Recreation, or Other.

#### Posture

Participants responded to 18 items asking them to indicate how frequently they were in the position displayed for that item. Their frequency responses were based on a 9-point scale with response categories ranging from “Never” to “More than 12 hours per day”. The images for the 18 items are described in Table 3 of the Results sections and the measure had a Chrobach’s alpha of 0.779. The images were adopted from several sources with six keyboard related images purchased from an online source [22]; four images showing postures with handheld devices came from a published Steelcase posture study [23]; and the remainder were purchased from another online image source [24].

#### Pain

Participants were asked to indicate the amount of pain or discomfort using a body map questionnaire with 16 different body regions [25]. The body regions were presented in a diagram format on the Qualtrics® survey and participants clicked on a body part to indicate the level of pain they were currently experiencing in that part. The body regions are listed in Table 5 of the Results section and participants responded on a 5-point scale: 1 - (None) No Pain/Discomfort; 2 - (Just Noticeable) Pain/Discomfort Does Not Restrict Activity; 3 - (Some Pain/Discomfort) Restricts Some Activity; 4 - (Moderate Pain/Discomfort) Restricts Most Activity); and 5 - (Intolerable Pain/Discomfort) Restricts All Activity. This questionnaire has been used widely in ergonomic research as it provides a clear reference to the body region the participant is to rate and can be done quickly and easily [26–28]. There was an overall Chrobach’s alpha of 0.894 for this measure.

#### Activity and stress

There were four questions created for this study that asked participants to indicate the number of days per week they engaged in moderate and vigorous exercise per week as well as the number of minutes per day they engaged in that experience. These were free response items. Participants also responded to the following item regarding stress: “Please choose the number (0-10) that best describes how much stress you have been experiencing in the last 30 days? 10 = Extreme Stress, 0 = No Stress.”

### Analyses

To identify differences in how long and where participants used their devices, repeated measure analysis of variance (RMANOVA) were conducted. These analyses were corrected for Sphericity (when necessary) using the Greenhouse-Geisser correction.

Data reduction analyses were conducted with the posture and pain responses using Principal Component Analysis (PCA), with Varimax orthogonal rotation using Kaiser Normalization. These analyses were conducted on the data obtained from all participants (*n* = 515) to determine the underlying structure of the items on the survey. Pairwise exclusion was used to adjust for those with missing data. All other analyses were performed on the participants with complete data (Posture: *n* = 512 and Pain: *n* = 506).

To identify predictors of participants’ pain, separate stepwise multiple regressions (SMR) were conducted for each of the components identified in the PCA for pain. The SMR included the predictor variables: Tech most, Tech use (for each device), Tech for (for each device), Tech no break (for each device), Hours tech (for each device), Sex, BMI, Positions (components from PCA). Due to the low number of participants in the gaming and “other” categories, these variables were not included in the SMR.

## Results

### Demographics

The demographics (academic level, ethnicity, race, sex, history of technology use, and gamer status) of the participants are as follows; Freshman (24.9%), Sophomore (21.2%), Junior 18.4%), Senior (21%), Senior+ (12.9%); Hispanic (18.3%), Non-Hispanic (80%); White (83.1%), Black (3.7%), Asian (10.7%), Hawaiian (1.4%), American Indian (3.9%); Male (66.8%), Female (30.5%), Other (.2%), Missing (2.5%); Historic User Yes (91.8%); Gamer Yes (25.4%). The average body mass index (BMI) of the participants was 24 (SD 4.2).

### Technology use

When asked to indicate the type of technology they used regularly (*Tech Use*), the highest number of participants reported use of Smartphone (*n* = 500) followed by Laptop (*n* = 494). The number of participants indicating they used Desktops and Tablets were 277 and 182, respectively. Eighteen participants indicated they used another type of technology, which included gaming consoles (*n* = 7), iPod® (*n* = 3), and a FitBit® (*n* = 1), with seven participants giving no response of the type of other technology they used. The Smartphone had the highest percent (64.0%) of participants indicating that this was the type of technology they used the most frequently (*Tech most*). This was followed by the Laptop (53.2%), Tablet (53.2%), and Desktop (46.4%).

### Time on technology

Table 1 provides descriptions of how participants used their technology along with daily activity levels. Specifically, the percent of time they used the technology for different activities, how many hours per day they used the technology, how long they worked on the technology without a break, and their current stress levels. For those variables where participants gave a specific value (e.g., Hours per day on the Smartphone), outlying (or impossible, e.g., 25 h per day) values were (i.e., identified through descriptive analyses [29]; Ludbrook, 2008) were considered missing values in the analyses and the descriptive statistics are presented below.

**Table 1:** Descriptive statistics for Time on Technology Metrics

	N	Median	Mean	SEM
Percent of time using technology for following activity (*Tech for*)				
School	512	50	49.4	1.1
Recreation	511	30	32.9	0.9
Work	504	0	9.2	0.7
Other	509	0	1.4	0.3
Hours per day on the … (*Hours tech*)				
Smartphone	485	3	4.10	0.1
Laptop	494	3.75	3.77	0.1
Desktop	441	1	1.10	0.1
Tablet	439	0	0.49	0.1
Other	48	0	0.74	0.3
Minutes without a break on the … (*Tech no break*)				
Laptop	473	60	78.6	3.0
Desktop	385	15	32.5	2.3
Smartphone	480	15	29.4	2.0
Tablet	367	0	14.0	1.3
Other	24	0	20.8	8.5

The percent of time they used technology for different activities (*Tech for*) was School (M = 49.4%), followed by Recreation (M = 32.9%), Work (M = 9.2%), and Other (M = 1.4%). An RMANOVA found that there was a significant effect for percent of time with different activities, *F* (2.2, 1087.9) = 619.29, *p* < 0.001, ${\upeta}_{\mathrm{p}}^2$ = 0.55, and pairwise comparisons indicated that all of means were significantly different from each other (all *p’s* < 0.001).

Also seen in Table 1 is that participants’ average hours of technology use per day (*Hours tech*) were highest for the Smartphone (M = 4.1, SEM = 0.1) and Laptop (M = 3.8, SEM = 0.1). The average use hours were lower for Desktop (M = 1.1, SEM = 0.1), Tablet (M = 0.49, SEM = 0.1), and other technologies (M = 0.74, SEM = 0.3). An RMANOVA was done with the four most frequently used technologies (i.e., Smartphone, Laptop, Desktop, and Tablet) and these results indicated there were significant differences in participants hourly uses of the technology, *F* (2.3, 971.6) = 285.8, *p* < 0.001, ${\upeta}_{\mathrm{p}}^2$ = 0.48. Pairwise comparisons indicated that the means of all four technologies were significantly different from each of the other devices (all *p’s* < 0.035).

The number of minutes participants reported using their technology without a break (*Tech no break*) is presented in Table 1. On average, the highest use without a break was highest for Laptop (M = 78.6, SEM = 3.0), followed by Desktop (M = 32.5 SEM = 2.3), Smartphone (M = 29.4, SEM = 2.0 respectively), and Other (M = 20.8, SEM = 8.5), and Tablet (M = 14.0, SEM = 1.3). RMANOVA indicates an effect of type of device on minutes without a break, *F* (2.6, 897.3) = 88.39, *p* < 0.001, ${\upeta}_{\mathrm{p}}^2$ = 0.21, and pairwise comparison found that all means were different at *p* < 0.001 except for Smartphone and Desktop (*p* = 0.66).

Given the low percentage of time the participants reported using technology for “Other” and the low number of the participants responding to the “Other” categories, these variables will not be included in the regression models.

Table 2 provides the descriptive statistics for the participants’ reported activity levels (both the levels and amount of exercise), the percent of their days spent in different positions, and the amount of stress they have experienced recently. The participants reported doing a moderate number of exercise and average of 5 days a week for 74 min per day. Regarding vigorous exercise, they reported doing it 2.5 days per week for 50.6 min per day. The participants spent most of their day sitting (51.9%), followed by Walking (20.8%), Standing (12.8%), and Labor (7.3%). Finally, participants reported a mean stress level of 6.0 (with 1 being low and 10 being high).

**Table 2:** Daily Activity Levels and Stress and descriptions of participants’ daily activity levels – both general and exercise

	N	Median	Mean	SEM
Level of Exercise…(Exercise)				
Moderate exercise—days per week	484	5	4.6	0.1
Moderate exercise—min per day	501	60	74.0	2.9
Vigorous exercise—days per week	491	2	2.5	0.1
Vigorous exercise—min per day	493	45	50.6	2.1
Percent of the day spent …(Day spent)				
Sitting	507	50.0	51.7	1.1
Walking	506	20.0	20.7	0.6
Standing	506	10.0	12.8	0.4
Labor	507	5.0	7.4	0.4
Amount of Stress	499	6	6.1	0.1

### Posture with technology

Table 3 provides participants responses regarding their posture when interacting with devices. The table provides a description of the posture, the image the participants saw, and descriptive statistics from the 9-point scale (treated continuously). Of the different postures, working on a laptop situated on a table in bent seated posture was found to be the most dominant (frequency and time in posture), followed by working on tablet situated on a table. The use of typical workstation, i.e., chair and table, were found to be the most common, followed by chair alone. Habitual postures, i.e., sitting/sleeping on a bed/sofa, were reported to be the least commonly adopted postures.

**Table 3:** Reported positions while interacting with devices and times in respective positions on a typical day (*n* = 507). Responses based on a 9-point scale: 1 - “Never” to 9 - “More than 12 hours per day”

	Median	Mean	SEM
Body positions			
Sitting at a desk, using a laptop, while talking on the phone (Elbow desk laptop)	4	4.11	0.07
Sitting at a desk, using a tablet, while leaning on the elbow (Elbow desk tablet)	4	3.50	0.07
Sitting at a desk, leaning over, and using a phone or tablet (Desk phone, tablet)	4	3.50	0.07
Sitting up straight (90° at waist, elbows, and knees) a desk, and using a phone or tablet (Desk Sit)	4	3.49	0.07
Relaxed posture (> 90° at waist, elbows, and knees) a desk, and using a phone or tablet (Desk laptop)	3	3.47	0.06
Reclining on the floor or in bed using a tablet (Floor/bed recline tablet)	3	3.22	0.07
Sitting on a chair with elbow on the table using a phone (Chair/table phone)	3	3.06	0.07
Sitting up straight (90° at waist, elbows, and knees) in a chair (Chair sit)	3	2.78	0.06
Reclining back in a bed using a laptop (Bed laptop-back)	2	2.59	0.07
Reclining on front in a bed using a laptop (Bed laptop-front)	2	2.30	0.07
Sitting in a chair with feet on ottoman using a laptop (Chair ottoman-laptop)	2	2.28	0.07
Standing at a standing desk (Desk Stand)	1	1.83	0.05
Hand positions			
From the top—Hands on keyboard with no deviation (Keyboard Hands-No Deviation)	4	3.78	0.07
From the top—Hands on keyboard with radial deviation (Keyboard-Radial deviation)	2	2.73	0.08
Side view—Hand at keyboard with no extension (Keyboard Hands-No Extension)	3	3.10	0.07
Side view—Hand at keyboard with wrist extension (Keyboard-Wrist extension)	3	2.99	0.08
From the top—Hand on mouse with ulnar deviation (Mouse-Ulnar deviation)	2	2.19	0.07
From the top—Hands on keyboard with ulnar deviation (Keyboard-Ulnar deviation)	1	1.60	0.06

Table 4 reports findings from the PCA analysis of the 18 positions, the rotated component matrix, and the percent variance explained for each component (bolded component coefficients denote component loadings). The PCA resulted in 17 items loading onto six components. The item Desk Laptop loaded equally onto three components and was thus removed from the analysis. Further, the item Keyboard-Wrist Flexion loaded onto two items and was place in the component that it loaded onto were it most logically fit (with Keyboard Hands-No Ext).

**Table 4:** Rotated component matrix from PCA using Varimax rotation and including % variance explained for the 18 positions. Bolded component coefficients denote component loadings

	Reclined	Lean/ Slouch	Wrists - Ulnar deviation	Desk posture	Wrists - Radial deviation	Wrists - Flexion
% Variance explained	22.74	9.70	9.19	8.05	6.54	5.54
Bed laptop-back	**0.75**	0.14	0.07	0.05	−0.01	0.05
Bed laptop-front	**0.69**	0.11	0.10	0.21	−0.08	−0.12
Chair ottoman-laptop	**0.66**	0.16	0.07	0.04	0.00	0.09
Floor/bed recline tablet	**0.51**	0.36	0.25	− 0.28	0.13	0.22
Elbow desk tablet	0.17	**0.78**	−0.04	0.07	0.03	−0.03
Elbow desk laptop	0.04	**0.70**	0.21	−0.31	0.11	0.06
Desk phone, tablet	0.08	**0.60**	0.23	0.07	−0.13	0.05
Chair/table phone	0.25	**0.53**	0.03	0.17	0.05	0.00
Mouse-Ulnar deviation	0.02	0.19	**0.69**	0.20	−0.01	−0.07
Keyboard-Ulnar deviation	0.33	−0.02	**0.66**	−0.02	−0.15	0.10
Desk Sit	−0.13	−0.10	0.24	**0.75**	0.17	0.21
Desk Stand	0.43	0.01	0.15	**0.61**	0.03	−0.01
Chair sit	0.26	0.38	−0.03	**0.58**	−0.08	−0.03
Keyboard Hands-No Dev	0.02	0.16	0.13	0.18	**0.85**	0.20
Keyboard-Radial deviation	0.08	0.20	0.42	0.12	**−0.69**	0.20
Keyboard Hands-No Ext	0.11	0.09	−0.05	0.14	0.09	**0.91**
Keyboard-Wrist Extension	0.11	0.21	0.64	0.13	0.19	**−0.48**

The first component explained 22.74% of the variance, had four items, and all showed someone reclining in some way (Recline)—Bed laptop-back, Bed laptop-front, Chair ottoman-laptop, and Floor/bed recline tablet. The second component contained four items—Elbow desk laptop, Elbow desk tablet, Desk phone, tablet, and Chair/table phone—all showing someone either leaning or slouching and explained 9.7% of the variance (Lean/Slouch). The third component (9.19% of the variance) was comprised of two associated with ulnar deviation of the wrists either with the mouse or keyboard (Mouse-Ulnar deviation and Keyboard-Ulnar deviation). The fourth component explained 8.05% of the variability and contained three items that show someone sitting or standing at a desk (Desk Sit, Desk Stand, and Chair Sit). The fifth and six components both contained items associated with hand posture at the keyboard: one component (explaining 6.54% variance) showed the hands at the keyboard from above—one with no deviation and the other with radial deviation. The other component shows hands at the keyboard from the side—again one with no deviation and the other with extension (explaining 5.54% of the variance). These have been named Wrists – Radial deviation and Wrist – Flexion respectively.

### Reported pain

The first section of Table 5 shows the descriptive statistics of participants’ responses to the items asking them to indicate their level of pain or discomfort on a 5-point scale (treated continuously). The second section of the Table 5 provides PCA analysis of the sixteen body parts for the items in three different components (see rotated component matrix). The first explained 40.6% of the variance and contained parts of the upper body not including the arms and hands (Upper Body)—Neck, Upper Back, Lower Back, Shoulders, Middle Back, and Eyes. The second component explained 10.3% of the variance and contained parts of the lower body (Lower Extremities)—Lower Legs, Thighs, Feet, Knees, Elbow, and Buttocks. The third component explained 7.5% of the variance and contained body parts in the distal upper extremities (Distal Upper Extremities)—Hands, Wrists, Lower Arms, and Upper Arms.

**Table 5:** Descriptive responses in descending order by Mean regarding reporting pain using a 5-point scale from 1 - (None) No Pain/Discomfort to 5 - (Intolerable Pain/Discomfort) Restricts All Activity (*n* = 506)

Component	Body Part	Median	Mean	SEM
UB	Lower Back	2	2.16	0.05
UB	Neck	2	2.02	0.04
UB	Shoulders	2	1.82	0.04
UB	Eyes	1	1.72	0.04
UB	Upper Back	1	1.67	0.04
UB	Middle Back	1	1.58	0.04
LE	Knees	1	1.55	0.04
DUE	Wrists	1	1.49	0.04
LE	Buttocks	1	1.41	0.04
DUE	Hands	1	1.39	0.03
LE	Feet	1	1.35	0.03
LE	Lower-Legs	1	1.23	0.03
DUE	Elbow	1	1.22	0.03
DUE	Lower Arms	1	1.20	0.03
LE	Thighs	1	1.20	0.02
DUE	Upper Arm	1	1.19	0.03
Rotated component matrix from PCA for the 16 body parts.				
	Upper Body (UB)	Lower Extremities (LE)	Distal Upper Extremities (DUE)	
% variance explained	40.6	10.3	7.5	
Neck	**0.81**	0.07	0.22	
Upper Back	**0.73**	0.19	0.17	
Lower Back	**0.73**	0.20	0.08	
Shoulders	**0.72**	0.18	0.19	
Middle Back	**0.63**	0.30	0.14	
Eyes	**0.46**	0.16	0.44	
Lower Legs	0.11	**0.77**	0.17	
Thighs	0.17	**0.72**	0.30	
Feet	0.28	**0.69**	0.09	
Knees	0.24	**0.65**	0.01	
Elbow	0.11	**0.58**	0.43	
Buttocks	0.34	**0.44**	0.37	
Hands	0.22	0.13	**0.79**	
Wrists	0.25	0.08	**0.78**	
Lower Arms	0.11	0.43	**0.69**	
Upper Arm	0.16	0.55	**0.56**	

Bolded component coefficients denote component loadings

### Predicting pain

Three stepwise multiple regressions SMR were conducted—one with each pain components as a criterion—to identify the variables that contributed to participants’ reported pain. The predictors used in the regression models were: Hours they used each technology (Tablet, Desktop, Laptop, Smartphone), Minutes they went without a break for each technology (Tablet, Desktop, Laptop, Smartphone), average score for each posture factor (Reclined, Lean/ Slouch, Wrists - Ulnar deviation, Desk posture, Wrists - Radial deviation, Wrists - Flexion), the percent of time they used any technology for different activities (Work, School, Recreation), Sex, BMI, the amount of stress they experienced, information about their exercise activities (Days per Week Moderate, Minutes per Day Moderate, Days per Week Vigorous, Minutes per Day Vigorous), and finally the percent of the day they spent Sitting, Standing, Walking, and in Labor.

Table 6 reports findings from the SMR for each of the different pain component criterion. For the Upper Extremity pain component, the final model had an adjusted variance explained (a.k.a., *R*^2^) of .13, and two variables contributed to this model. The Percent Time of Technology Use for School explained 8% unique variance and was negatively related to Upper Extremity pain. The Reclined Position component explained 5% unique variance and was positively related to Upper Extremity pain.

**Table 6:** Findings from stepwise multiple regression for the three pain components for the predictors^a^

	Standardized Coefficients		Sig.	Δ *R*^2^	*R*^2^
	Beta	t			
Upper Extremity					
Percent Time Use/School	−0.26	−2.96	0.00	0.08	
Reclined Posture Component	0.23	2.60	0.01	0.05	0.13
Upper Body					
Amount of Stress	0.29	3.43	0.00	0.16	
Lean/Slouch Posture Component	0.28	3.26	0.00	0.07	
Reclined Posture Component	0.18	2.28	0.03	0.03	
Wrists Ulnar Deviation Component	0.16	1.99	0.05	0.03	0.28
Lower Body					
Reclined Posture Component	0.23	2.64	0.01	0.08	
Minutes No Break/Tablet	0.24	2.79	0.01	0.05	
Amount of Stress	0.19	2.26	0.03	0.04	0.17

^a^Predictors: Hrs each tech (Tablet, Desktop, Laptop, Smartphone); Min. without break each tech (Table

t, Desktop, Laptop, Smartphone); avg. each posture factor (Reclined, Lean/ Slouch, Wrists - Ulnar deviation, Desk posture, Wrists - Radial deviation, Wrists - Flexion), percent time used tech per activities (Work, School, Recreation); Sex; BMI; stress; exercise (Days per Week Moderate, Minutes per Day Moderate, Days per Week Vigorous, Minutes per Day Vigorous); percent day spent Sitting, Standing, Walking, and in Labor

For the Upper Body component, the final model had an adjusted *R*^2^ of .28 indicating it explained 28% of the variance. As seen in Table 5, there were four variables that contributed to this model, with the Amount of Stress contributing the most unique variance (16% unique variance). Lean/Slouch Posture Component contributed 7% unique variance and the Reclined Posture Component and Wrists Ulnar Deviation Component both contributed 3% unique variance to Upper Body pain.

The third pain component, Lower Body, had three variables that uniquely and positively contributed to the total overall variability in this model (total variance explained was 17%). For this model, Reclined Posture explained 8% unique variance and Minutes on the Tablet with no break explained 5% unique variance. The third variable was Amount of Stress and this explained 4% unique variance.

## Discussion

This study explored technology use among college students and its relation to their reported pain with a particular interest on the use of smartphones. Specifically examined was devices type, device usage, and posture and any connection between these variables and reported pain level among this population. School (49.4%) and recreation (32.9%) were the activities they used technology for the most and they used their smartphone (4.10) and laptop (3.77) for the highest number of hours. However, it was the laptop that they would use the longest without a break (78.6 min). The focus of this study was particularly on identifying whether, and if so, how the rapid and ubiquitous adoption of handheld devices (e.g., smartphones and tablets) by college students impacted their experience of pain. To explore this, we conducted stepwise multiple regressions—if our results had corresponded with previous results, we would expect to find that variables associated the type of device (specifically the use of tablets and laptops) would contribute the most variability to the reported pain in the upper body and upper extremity pain components. If our expectation that the pervasive use of smartphones was a concern, we would expect variables associated the use of smartphones to contribute the most variability to reported pain. However, we found neither. Indeed, tablet use (i.e., minutes of tablet use with no break) was the only predictor that contributed to reported pain and this was for the lower body component. Further, although the minutes of tablet use with no break significantly contributed to the model, it was only 5% of the overall explained variance of 17% for this model.

Our results instead show that posture components and stress more consistently contributing to students’ reported pain. This is particularly seen in the model explaining the Upper Body reported pain. This model explained 28% of the variance in reported pain and Stress explained 16% of that variance. Further, three different posture components—Lean/Slouch, Reclined, and Wrist Ulnar Deviation—explained the additional 13% (7, 3%, & 3% respectively). Further, in this study, for all three body groups (Upper extremity, Upper body, and Lower body), the Reclined Posture Component contributed significantly to reported pain—with more time in these postures resulting in more pain (see Table 6). Students generally experienced more pain in the Upper Body regions (see Table 5) than the other two components.

There were interesting differences between the predictors of reported pain for three body groups. For instance, for the Distal Upper Extremities (hands, wrists, upper & lower arms), the Percent Time Use/School was *negatively* related to pain, indicating that the more they used any device for School, the less pain they experience in this body region. This result is difficult to interpret but could be due to generally lower pain ratings in this body region. It could also indicate that this measure is not sensitive for these body regions, particularly in this population (i.e., primarily young, and healthy participants) particularly given that previous studies using the Body Discomfort Diagrams have primarily focused on working adults [26–28].

Another interesting finding was the contribution of stress to the variability in reported pain for the Upper Body (Neck, Upper/Lower/Middle back, Shoulder, and Eyes). As seen in Table 6, Stress accounted for over half of the variability explained by the model (16% out of 28%), while posture components (i.e., Lean/Slouch, Reclined, and Wrist Ulnar Deviation) accounted for the rest. That stress would be related to pain in the Upper Body is not surprising given that many tend to tighten their shoulders when stressed and is consistent with the literature on findings related to neck and shoulder pain prevalence among college students [30–34]. However, the fact that stress contributes to the Lower Body as well suggests that in this population, stress management maybe as important for mitigating pain as ergonomic interventions that impact posture and device use. Indeed, given that their average stress level was 6.1 on a scale with 0 = No Stress and 10 = Extreme Stress, the amount of stress these students are experiencing combined with less experience managing stress could be an area for future investigation.

Generally, college students still reported significant portions of their day in traditional desk and chair work positions with laptops and desktops. However, the use of alternate postures such as floor, bed and chair without desk were also very common along with the multitude of postures used with smartphones that are less constrained due to their weight, size, and heat output. The result of these postures and durations with respect to reported discomfort was concentrated in the neck, back, and eyes. Albeit low levels of relative discomfort as is often typical with a sample population of young & mostly healthy adults such as that reviewed by Towne et al. in a previous paper on this same college student cohort that included a look at sex, sleep and race/ethnicity [35]. For future research, “floor/bed phone” as an option should be considered as students pointed out this was missing and certainly a common activity for them.

Although it is surprising that the number of hours using a device did not contribute at all to the reported pain and that the number of minutes without a break only contributed to one component for one device (the tablet), these results are somewhat promising for ergonomic interventions with this population. The results essentially indicate that, regardless of device, ergonomic interventions that focus on improving posture and facilitating stress management may reduce the likelihood of pain. Although clearly the reported pain is not high on average for this group, it does exist and that is concerning in and of itself given that these are young and relatively healthy people. Further, as mentioned previously, their stress level is also somewhat concerning. Therefore, these results suggest that posture and stress management interventions may be effective regardless of device and thus, as new technologies are introduced and adopted, the intervention methodologies may not need to be remarkably different.

In general, individuals who spent more time in sedentary postures reported more pain and would therefore benefit from behavioral modifications that encourage different postures throughout the day. This is timely for college students, given major universities have transitioned, in many cases, to remote learning since COVID-19. This introduction of significant shifts in the students’ learning environment, including the students’ ability to access optimal ergonomic set ups (e.g., devices and related equipment), may lead to more lasting technology-use behaviors. For those already in office environments in major industries that have shifted to remote work for months, potentially longer, this is also an important consideration. For example, it is unlikely that all will have distinct and necessary office space in their home that might help facilitate a more ergonomic set up. Furthermore, having appropriate equipment (e.g., ergonomic keyboards, monitors) to utilize may also take additional resources unavailable for many. Thus, the availability of proper equipment and space for both college students, but also those already in industries previously relying on office workspaces prior to COVID-19 clearly presents a challenge. Anecdotally speaking, since COVID-19, we have seen an increase of both students and adults working from home-based workstations such as couches, beds, and kitchen counters. If this is to become more common than before COVID-19, additional work may need to be done with remote workers to evaluate risk levels and then if needed, provide evidence-based interventions. The potential cost of resources needed to facilitate an ergonomic-like environment within one’s home, potentially including the added cost of additional square footage (especially for already space-restricted apartment homes) and additional resource cost of purchasing equipment, may lead to disparities across income, a known social and structural determinant of health inequities [36]. By extension, the known health issues stemming from inadequate ergonomic set ups and device interactions may disproportionately affect those already at-risk of health inequities. Indeed, an algorithmic approach Pierson et al [37] attempted to explain the pain disparities in underserved populations and how they might apply to improved treatment approaches for clinicians. However, this variability has not been researched with respect to pain disparities associated with ergonomic issues or in college students. Therefore, studies exploring these potential disparities are recommended for future research. Furthermore, employers who either do not recognize the importance of ergonomics, during temporary remote working conditions, or those who cannot support the additional costs involved in supplying appropriate office-like environments during remote work under the pandemic may also contribute to these potential disparities.

With a tech-focused society and growing rates of technology use (smartphones and laptops), efforts are needed to ensure pain is deferred or delayed until later years if the productivity of this workforce is to be preserved. Our team believes that although technology induced inactivity has been part of the struggle for information age workers to move and remain healthy, it is also technology that is most likely to provide smart solutions derived from “precision ergonomics” that will supplement the population level solutions of one approach for all that have been most common up until now. This concept is discussed as “agile science” by Patrick et al. in their discussion of technology and health finally coming together for the good of people on a large scale [38].

### Limitations

One of the major limitations of this study is the use of surveys to assess the constructs of interest. Self-report data may not be as reliable as observational data and at the same time, they may allow for evaluation of constructs with a larger sample. The accuracy of self-reports is inherently challenged due to recall bias and social desirability. For example, either of these could result in some respondents reporting higher or lower levels of screen time or time in specific postures. That said, given the confidential nature of survey responses in this online survey it may be that respondents were more likely to report more accurate estimates than one might otherwise. For example, if it had been an in-person survey where one’s instructors or advisors were aware of their responses tied to their names, this could increase the potential for bias associated with responses tied to social desirability. Thus, the confidential nature of the survey was a major strength in this regard. Another limitation is associated with the sample. Given that the data were taken from a single, albeit large, STEM-focused department in one of the largest universities in the US, it is not likely to be generalizable to the entire student body. However, this was not an expectation, given variation in demographic composition within and between university settings. Even with these limitations, the findings have important implications regarding the importance of understanding ergonomic risk factors in this population.

## Conclusion

As we seek to further enhance capabilities of hand-held devices (primarily smartphones), we need to understand the increase in exposure to device use and the transition to unhealthy postures that they permit via macro design features. Further, the recent addition of streaming services has added to the overall viewing time by handheld device users. If the same device is used for pleasure viewing like binge watching television shows and completing homework and other assignments, the human interface for control and display will continue to grow in importance to our health outcomes. Given that the goal is to prevent musculoskeletal injuries and illness among students and adults using these devices, our findings suggest we many need to reconsider our approach. As noted above our team believes, as others have tested, that the use of these same devices to prompt, encourage, and nudge proper behaviors, including breaks from their use and proper ergonomics, are both feasible and the most likely path toward better health outcomes for students [39, 40]. The understanding we provide here should help device designers and programmers better plan for user exposure to their software, apps, accessories, tools, cases and devices within the heavy use crowd of college age students.

## Data Availability

The data that support the findings of this study are protected in accordance with the Texas A&M University Institutional Review Board for the current study, and so are not publicly available.
